# Zicam-Induced Damage to Mouse and Human Nasal Tissue

**DOI:** 10.1371/journal.pone.0007647

**Published:** 2009-10-30

**Authors:** Jae H. Lim, Greg E. Davis, Zhenshan Wang, Vicky Li, Yuping Wu, Tessa C. Rue, Daniel R. Storm

**Affiliations:** 1 Department of Otolaryngology-Head and Neck Surgery, University of Washington, Seattle, Washington, United States of America; 2 Department of Pharmacology, University of Washington, Seattle, Washington, United States of America; 3 Department of Biostatistics, University of Washington, Seattle, Washington, United States of America; Duke Unviersity, United States of America

## Abstract

Intranasal medications are used to treat various nasal disorders. However, their effects on olfaction remain unknown. Zicam (zinc gluconate; Matrixx Initiatives, Inc), a homeopathic substance marketed to alleviate cold symptoms, has been implicated in olfactory dysfunction. Here, we investigated Zicam and several common intranasal agents for their effects on olfactory function. Zicam was the only substance that showed significant cytotoxicity in both mouse and human nasal tissue. Specifically, Zicam-treated mice had disrupted sensitivity of olfactory sensory neurons to odorant stimulation and were unable to detect novel odorants in behavioral testing. These findings were long-term as no recovery of function was observed after two months. Finally, human nasal explants treated with Zicam displayed significantly elevated extracellular lactate dehydrogenase levels compared to saline-treated controls, suggesting severe necrosis that was confirmed on histology. Our results demonstrate that Zicam use could irreversibly damage mouse and human nasal tissue and may lead to significant smell dysfunction.

## Introduction

As one of the five senses, the ability to smell plays a crucial role in defining the quality of life. Indeed, loss of sense of smell or anosmia can have detrimental consequences as our ability to detect and process noxious chemosensory stimuli is impaired [1]–[3]. For instance, failure to detect smoke from a house fire while asleep is one such life-threatening situation. Because the sense of smell is intimately linked to gustatory function, smell dysfunction can negatively impact our sense of taste [4], [5]. Hence, even a small loss or alteration of smell can significantly disrupt one's quality of life. Nonetheless, the etiology and mechanism underlying the development of olfactory dysfunction is unclear.

One possible cause for the development of smell dysfunction is the use of various intranasal medications. Numerous intranasal drugs are available to treat various nasal disorders, including sinusitis, allergic rhinitis and nasal congestion. Although the safety and efficacy of the majority of these agents are well known, their effects on olfaction are not established. Moreover, some intranasal agents classified as “homeopathic” are marketed to treat common nasal disorders such as symptoms associated with the common cold. These agents are gaining popularity with consumers despite the lack of scientific data on their safety and efficacy [6]. Given the importance of the sense of smell in an individual's quality of life, the effects of various intranasal drugs – both conventional and “homeopathic” on olfaction need to be determined.

In this study, we examined the short-term and long-term effects of several commonly used intranasal agents using mouse and organotypic cultures from human nasal tissue. Specifically, we tested saline, Afrin (Schering-Plough, Kenilworth, NJ), Nasacort (Sanofi-Aventis, Bridgewater, NJ), lidocaine (Hospira, Inc., Lake Forest, IL) and epinephrine (Hospira, Inc., Lake Forest, IL), which are frequently utilized by physicians to treat various nasal disorders. Additionally, we tested one of the “homeopathic”, zinc-based intranasal agents, Zicam (Matrixx Initiatives, Inc., Phoenix, AZ) as it has been previously implicated in smell dysfunction [7], [8]. We used a combination of electrophysiological, biochemical and behavioral assays to determine whether application of these intranasal preparations leads to the development of nasal dysfunction.

## Results

### Odorant-induced activity of mouse olfactory sensory neurons following intranasal agent administration

**Figure 1:**
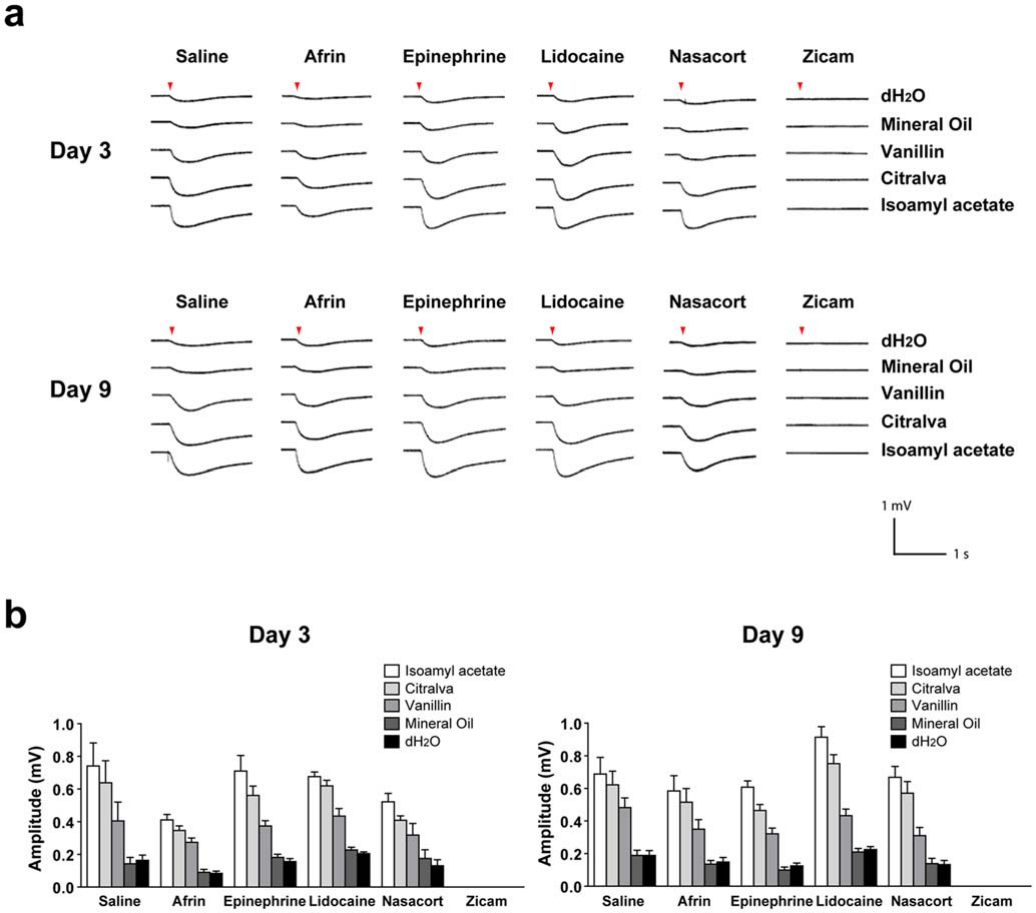
Odorant detection defects in the MOE of Zicam-treated mice. (a) Odorant-stimulated EOG responses from mice treated with either saline, Afrin, epinephrine, lidocaine, Nasacort or Zicam, 3 and 9 days following intranasal administration. Red arrowhead indicates the time at which the odorant was delivered to the MOE. (b) Summary of the mean EOG amplitudes in response to odorants on day 3 and 9 following intranasal agent administration. Only Zicam-treated MOE failed to elicit odorant-stimulated EOG response (n = 5 for all groups).

To determine the effects of various intranasal agents on the functional properties of olfactory sensory neurons (OSN), we performed electro-olfactogram (EOG) analysis on mouse main olfactory epithelium (MOE) 3 and 9 days after intranasal administration of either saline, Afrin, Nasacort, epinephrine, lidocaine or Zicam. Following odorant stimulation, the activation of OSN *in vitro* was observed in all animal groups except for those treated with Zicam (**Figure 1a,b**). Specifically, Zicam-treated MOE failed to elicit any measurable response from the OSN following odorant stimulation. We also consistently observed atrophic MOE in Zicam-treated animals as compared to the animals treated with other intranasal agents (**Figure S1,S2**).

### AC3, β-tubulin and OMP expression following intranasal agent administration

**Figure 2:**
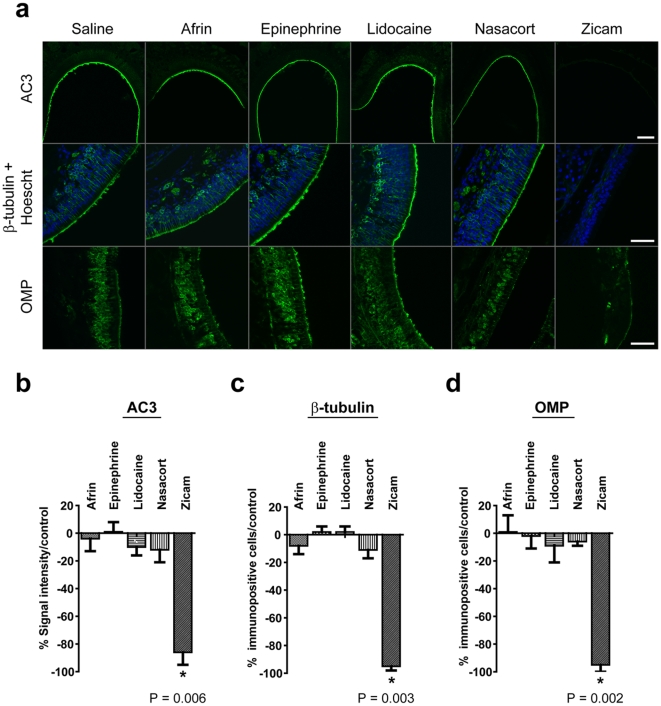
Expression of AC3, β-tubulin and OMP following various intranasal agent administrations. (a) Immunofluorescence of mouse MOE stained with antibodies specific for AC3, β-tubulin, and OMP. Hoechst counterstain is shown in blue. Scale bar, 10 µm for AC3; 20 µm for β-tubulin and OMP. (b) Analysis of AC3 immunofluorescence signal intensity in MOE (Afrin, Epinephrine, Lidocaine, Nasacort, Zicam, *p = 0.006; n = 4 for all groups). (c) Analysis of β-tubulin immunopositive cells in MOE (Afrin, Epinephrine, Lidocaine, Nasacort, Zicam; *p = 0.003; n = 4 for all groups). (d) Analysis of OMP immunopositive cells in MOE (Afrin, Epinephrine, Lidocaine, Nasacort, Zicam, *p = 0.002; n = 4 for all groups). All tested groups were normalized to saline-treated controls.

We also examined the MOE for the expression of several proteins expressed in OSN. In all MOE examined, except those treated with Zicam, immunofluorescence staining demonstrated robust expression of AC3 (adenylyl cyclase 3) in the olfactory cilia, neural-specific β-tubulin and OMP (olfactory marker protein) in the cell bodies and processes of OSN (**Figure 2a**). Zicam-treated MOE showed significant reductions in the expression of above biochemical markers. Specifically, 9 days after Zicam treatment, there was an 86% reduction in AC3 signal intensity (p<0.01; **Figure 2b**), and 95% decrease in both β-tubulin (p<0.005; **Figure 2c**) and OMP (p<0.005; **Figure 2d**) immunopositive cells compared to the saline-treated MOE. The intensity of AC3 immunofluorescence signal and the number of immunopositive β-tubulin and OMP cells were not statistically significant in MOE treated with either Afrin, Nasacort, lidocaine or epinephrine compared to the saline administered group (**Figure. 2b,c,d**)

### Long-term suppression of odorant-induced activity of OSN by Zicam treatment

**Figure 3:**
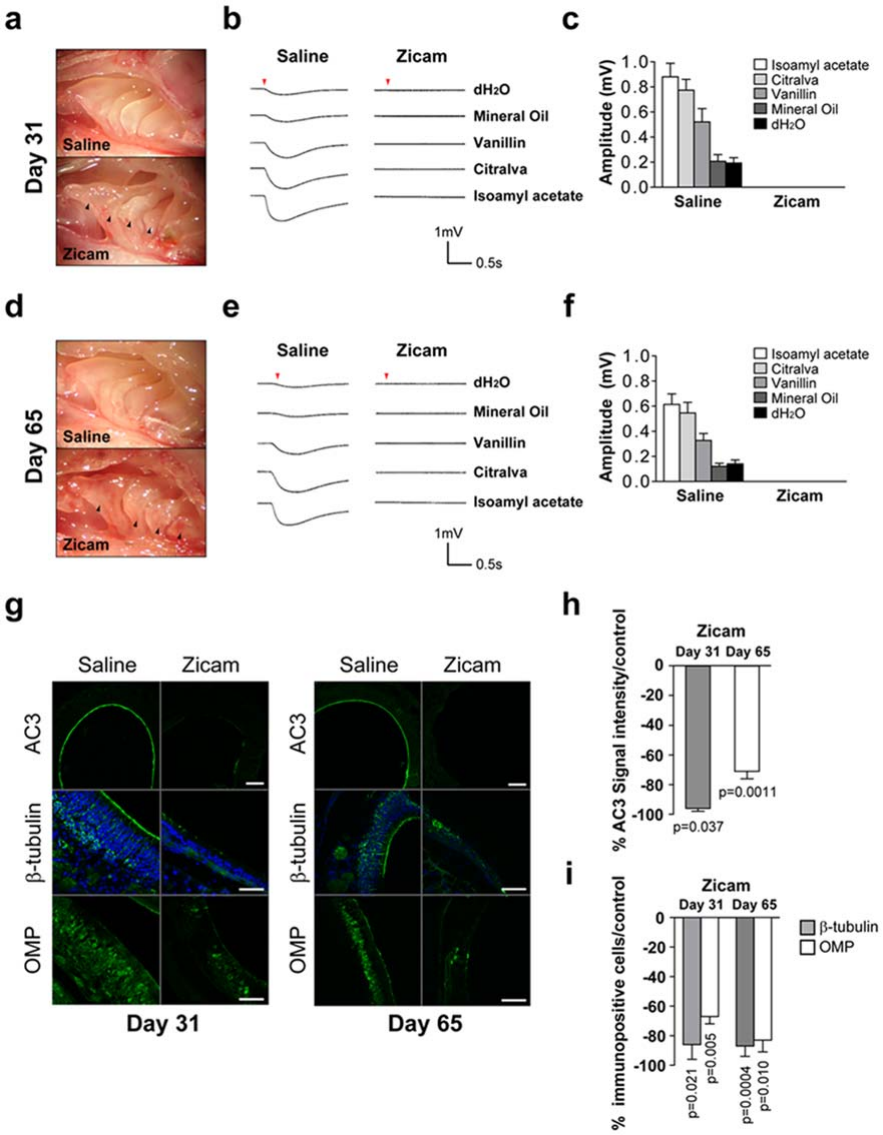
Long-term odorant detection defects in the MOE of Zicam-treated mice. (a) Appearance of MOE on day 31 in saline or Zicam-treated mice. Black arrowheads depict significant atrophy in endoturbinates of Zicam-treated MOE. (b) Odorant-stimulated EOG responses from saline or Zicam-treated mice, 31 days following intranasal administration. Red arrowhead indicates the time at which the odorant was delivered to the MOE. (c) Summary of the mean EOG amplitudes in response to odorants on day 31 in saline or Zicam-treated mice. Zicam-treated MOE failed to elicit odorant-stimulated EOG response (n = 4, saline; n = 5, Zicam). (d) Appearance of MOE 65 days after intranasal administration of either saline or Zicam. (e) Odorant-stimulated EOG responses from mice treated with either saline or Zicam, 65 days following intranasal administration. (f) Summary of the mean EOG amplitudes in response to odorants 65 days following either saline or Zicam treatment (n = 5 for all groups). (g) Immunofluorescence of mouse MOE stained with antibodies specific for AC3, β-tubulin, and OMP 31 and 65 days after saline or Zicam treatment. Hoechst counterstain is shown in blue. Scale bar, 10 µm for AC3; 20 µm for β-tubulin and OMP. (h) Analysis of AC3 immunofluorescence signal intensity in MOE of Zicam-treated mice normalized to saline-treated controls (p = 0.05, day 31; p<0.005, day 65; n = 4 for all groups). (i) Analysis of β-tubulin (p<0.05, day 31; p<0.0005, day 65) and OMP (p<0.01, day 31; p<0.05, day 65) immunopositive cells in the MOE treated with Zicam normalized to saline-treated controls (n = 4 for all groups).

Given that MOE maintains regenerative capacity following injury [9], [10], we investigated the possibility of functional recovery in the MOE following Zicam treatment. We performed EOG recordings 31 and 65 days after intranasal administration of Zicam in mice. Odorant stimulation failed to elicit any response from the OSN at either of these time points (**Figure. 3b,c,e,f**). Again, we observed significant atrophy of the MOE in all Zicam-treated mice, and often had difficulty capturing the baseline electrical signal (**Figure 3a,d** **; Figure S2**). Furthermore, we examined the expression of AC3, β-tubulin and OMP by immunofluorescence. We observed significant reductions in the expression of these markers on days 31 and 65 following Zicam administration (**Figure 3g,h,i**). Specifically, the intensity of the AC3 signal was reduced 96% and 71% in Zicam-treated MOE on days 31 and 65, respectively, as compared to the saline-treated MOE (**Figure 3h**). In addition, Zicam-treated MOE showed 87% and 67% reductions in β-tubulin and OMP immunopositive cells on day 31, respectively, as compared to the saline-treated MOE (**Figure 3i**). Finally, on day 65 following Zicam treatment, we observed 87% and 83% reductions in β-tubulin and OMP immunopositive cells, respectively (**Figure 3i**), as compared to the saline-treated controls. The data collectively indicate a remarkable damage to the olfactory epithelium and a significant loss of regenerative capacity in Zicam-treated mice.

### Deficits in olfaction following Zicam treatment

**Figure 4:**
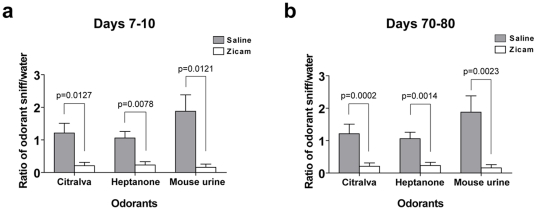
Olfactory behavior deficits in Zicam-treated mice. (a) Olfactory habituation assay 7–10 days following saline or Zicam intranasal treatment. The comparison of the ratio of the number of times the mouse sniffed an odorant-soaked cotton swab to the number of times it sniffed a water-soaked cotton swab on initial exposure is an indication of the ability of the animal to detect a specific substance. Cotton swabs were laced with 50 µl of citralva (10 µm), heptanone (50 µm) or mouse male urine (50-fold dilution). Significant differences are noted for the ability of Zicam-treated (n = 8) and saline-treated mice (n = 8) to detect citralva (p<0.05), heptanone (p<0.01) and male urine (p<0.05). (b) Olfactory habituation assay 70–80 days following saline or Zicam intranasal treatment. Again, significant differences are observed for the ability of Zicam-treated (n = 8) and saline-treated mice (n = 8) to detect citralva (p<0.0005), heptanone (p<0.005) and male urine (p<0.0005).

Although our EOG findings demonstrated the lack of odorant-stimulated electrophysiological response in Zicam-treated MOE, it is nonetheless an *in vitro* preparation and requires further validation. Therefore, we performed the olfactory habituation assay to detect potential behavioral deficits in olfaction in Zicam-treated mice. Mice failed to recognize novel odorants approximately 1 week after bilateral intranasal administration of Zicam (**Figure 4a**). Similar behavioral phenotype was again observed approximately 2 months following Zicam administration, suggesting the lack of recovery of function (**Figure 4b**). These mice behaved in similar manner to anosmic AC3^−/−^ mice [11].

### Elevated release of lactate dehydrogenase (LDH) levels in human nasal explants following Zicam application

**Figure 5:**
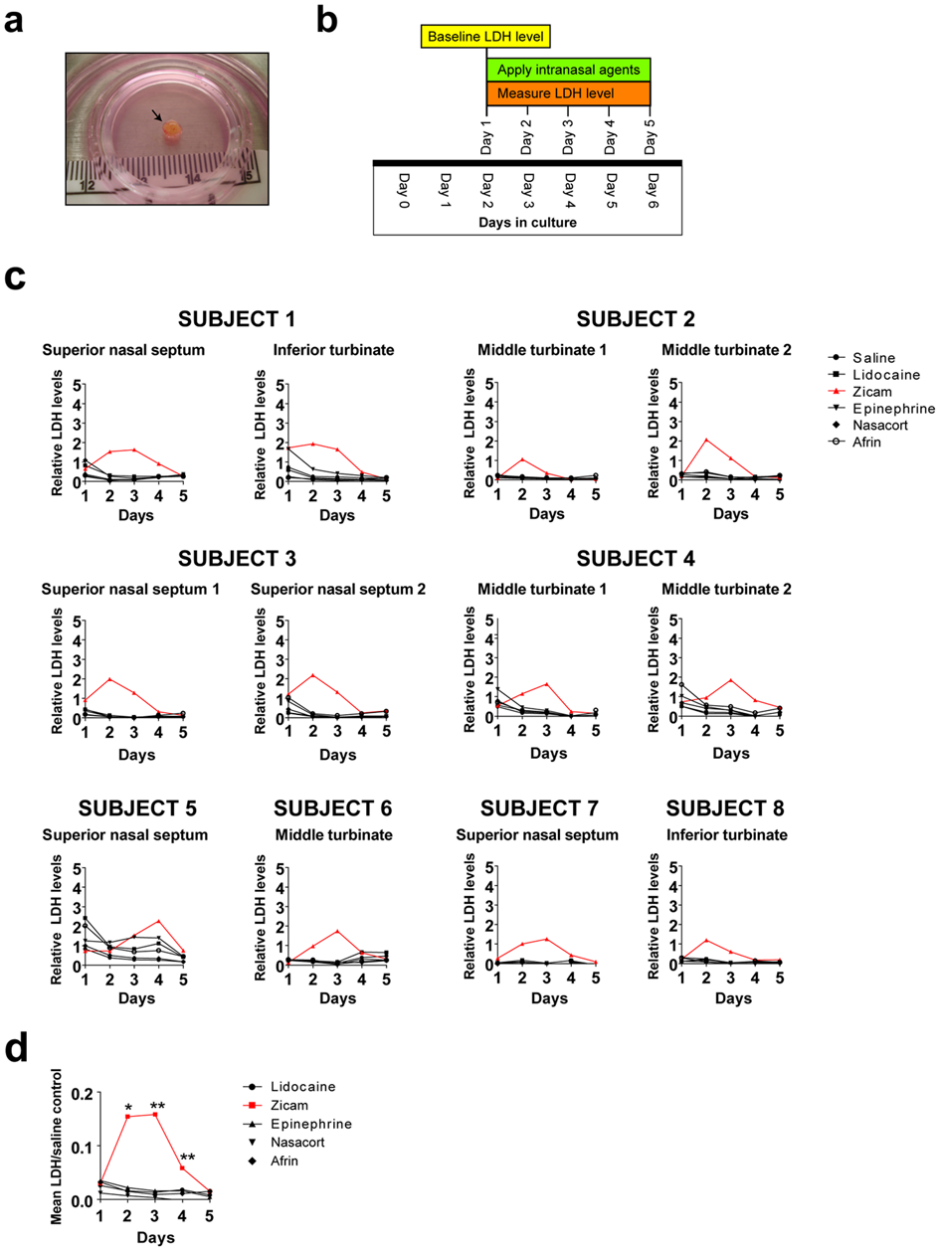
LDH elevation in Zicam-treated human nasal explants. (a) Organotypic tissue culture system setup for human nasal explants. Black arrow depicts newly harvested human middle turbinate tissue. (b) Graphical overview of the LDH assay. (c) Analysis of relative LDH levels from human nasal explants treated with either saline, Afrin, lidocaine, Nasacort, epinephrine or Zicam from various nasal regions over 5 days. LDH levels for each group are normalized to the control growth medium. (d) Summary of mean LDH levels from lidocaine, Zicam (day 2, *p<0.02; day 3,**p<0.002; day 4, **p<0.002), epinephrine, Nasacort and Afrin-treated human nasal explants normalized to saline-treated controls. All LDH levels other than the Zicam day 2, 3 and 4 did not reach statistical significance.

Our experiments in mice indicated the likelihood of developing significant olfactory dysfunction following Zicam treatment. Because a mouse study alone cannot adequately predict the biological effects of intranasal agents on human nasal tissue, we established an organotypic tissue culture system with human nasal explants (**Figure 5a**). Eight subjects met inclusion criteria. These subjects represent a wide spectrum of pathologic diseases and pre-operative olfactory function (**Table 1**).

**Table 1:** Characteristics of Human Subjects.

Age (range, mean)	20–58, 44.6
Male	4
Female	4
UPSIT (range, mean)	13–39, 27
LM CT (range, mean)	1–24, 5.4
SNOT (range, mean)	0–3.15, 1.50
Indication for surgery (N)	
Chronic sinusitis	5^a^
Pituitary tumor	1^b^
Nasal congestion	2^c^
LM CT, Lund-Mackay staging system; SNOT-20, Sino-Nasal Outcomes Test; UPSIT, University of Pennsylvania Smell Identification Test).	

a Functional endoscopic sinus surgery.

b Transnasal endoscopic approach to the pituitary tumor removal.

c Inferior turbinate submucosal resection.

We measured extracellular LDH levels following application of various intranasal agents directly onto the human nasal explants as a measure of cellular damage (**Figure 5b**). The results demonstrated a rapid and significant elevation of LDH levels within the first few days of Zicam application when compared to the LDH released in the saline control group (**Figure 5c,d**). Even after adjusting for multiple comparisons, of all the intranasal agents tested, only the Zicam treated nasal tissue showed statistically significant elevated LDH levels compared to saline control (**Figure 5d**). The finding was consistent in all nasal anatomic regions tested (i.e., superior nasal septum, middle turbinate and inferior turbinate). In contrast, human nasal explants treated with all other intranasal agents showed rapid reduction in LDH level followed by stabilization suggesting little or no cytotoxicity on the nasal tissue (**Figure 5c,d**).

### Zicam mediated cytotoxicity in human nasal explants

**Figure 6:**
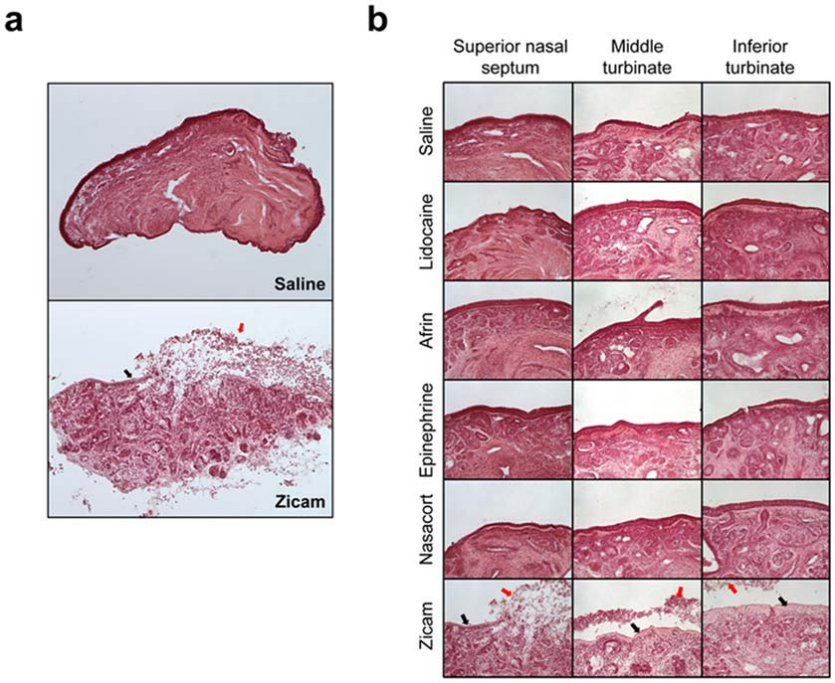
Cellular necrosis in Zicam-treated human nasal explants. (a) Hematoxylin and eosin (H&E) stained human nasal explants from superior nasal septum following saline or Zicam application. (b) H&E stained human nasal explants from various nasal regions treated with either saline, lidocaine, Afrin, epinephrine, Nasacort or Zicam. Black arrows depict dead epithelial cells. Red arrows indicate basal surface of the remaining epithelium. Note the infiltration of inflammatory cells and severe necrosis in Zicam treated-nasal explants.

The elevation in extracellular LDH levels following Zicam treatment suggested cell death mediated by necrosis. Histological evaluation of Zicam-treated human nasal tissue indeed confirmed the presence of significant necrosis with the destruction of olfactory epithelium and subepithelial structures (**Figure 6a,b** **; Figure S3**). In contrast, nasal explants treated with other intranasal agents showed overall healthy morphology with little histological evidence of cytotoxicity (**Figure 6b**). Overall, cellular damage to human nasal tissue induced by Zicam was severe and was observed in every tissue that we examined for this study (**Figure S3**).

## Discussion

The present study demonstrated that Zicam treatment to both mouse and human nasal tissue induced significant cellular damage. In contrast, treatment with either saline, Afrin, Nasacort, lidocaine and epinephrine did not cause statistically significant tissue damage or olfactory dysfunction. The cytotoxic effects of Zicam were especially profound in the olfactory neuroepithelium. The resultant death of olfactory sensory neurons (OSN) in the MOE contributed to a long-lasting and apparently irreversible olfactory dysfunction in the mouse. Despite the ability of MOE to replace injured OSN via differentiation of stem cells, we found no evidence of functional recovery after two months. In fact, histological analysis of the mouse MOE about two months after Zicam treatment showed complete or near-complete loss of the epithelium and submucosa (**Figure S2**). In addition, significant necrosis of the epithelial and subepithelial structures occurred in human nasal explants following Zicam application. Thus, the loss of stem cells is feasible and could explain the lack of recovery of olfactory function in our mouse model. Collectively, the data illustrate that Zicam treatment can damage mouse and human nasal tissue with resultant loss of olfaction.

Our findings contradict a previous report on the effects of intranasal treatment of Zicam in mice [12]. The study showed that intranasal administrations with smaller volumes (i.e., 2 µl or 8 µl) of Zicam did not significantly compromise the integrity of olfactory epithelium and olfactory function. However, we find several caveats in their methodology and interpretation of the data. First, it is difficult to inject small volumes (<10 µl) of intranasal agents in unanesthetized mice, even if they are restrained. Based on our experiences with intranasal dye injections, such small volumes did not distribute thoroughly in the MOE, and sometimes missed the MOE entirely. Therefore, we performed slow, controlled intranasal injections in anesthetized mice with a targeted volume of about 15 µl that consistently distributed throughout the MOE. Since our study aimed to determine the role of various intranasal agents on olfaction, such a technique is necessary in order to ensure the effective delivery of the agents to the MOE. While this may reflect a higher dosage (i.e., based on nasal cavity volume alone) than that recommended for human use, it is difficult to determine the effects of intranasal agents on olfaction if smaller volumes are used in an *in vivo* mouse model system. Furthermore, we used the same volume for all other intranasal agents (i.e., saline, Afrin, Nasacort, lidocaine and epinephrine), and none of these drugs showed any significant signs of cytotoxicity to either mouse or human nasal tissue, confirming their safety profile.

Second, Slotnick and colleagues utilized anterograde tracing with wheat germ agglutinin horseradish peroxidase to determine the integrity of olfactory epithelium [12]. They suggested that despite the noticeable injury to the olfactory epithelium following Zicam treatment, the effects are transient and that recovery of olfaction is possible. However, the study failed to show any statistical analysis of their findings and lacked direct evidence of functional recovery at the level of olfactory epithelium. Notably, the observation that disruption of olfactory circuitry occurred with Zicam treatment (even with lower doses) in the mouse model should be worrisome because human OSN occupies a much smaller area in the nasal cavity than mice OSN [13]. Therefore, the risk of developing smell dysfunction could be significant if Zicam contacts the olfactory neuroepithelium in the human nasal cavity. Although most studies have focused on the effects of Zicam on olfactory neuroepithelium, and hence smell dysfunction, we emphasize that the cytotoxic effects of Zicam encompass a wider region of the nasal tissue. These include many subepithelial structures such as mucous glands, capillary networks and trigeminal nerve fibers. While these components do not directly contribute to smell dysfunction, they are critical to maintaining healthy nasal function [14], [15]. Presently, the long-term consequences of damage to these subepithelial structures are unknown.

Many studies have demonstrated the cytotoxic effects of zinc cation on various cell types, including cultured cortical neurons [16]–[20], neuronal PC12 cells [21] and olfactory epithelium [22]–[25]. The mechanism underlying zinc-mediated cell death appears to be oxidative necrosis [16], [21]. As demonstrated in our study, human nasal explants undergo rapid necrosis as evidenced by elevated extracellular LDH levels within first few days of treatment with Zicam (**Figure 5** **,** **6**). We have also performed the TUNEL (terminal dUTP nick-end labeling) assay with mouse MOE and did not find much apoptosis following Zicam administration (data not shown). Therefore, necrosis is likely to be the key mechanism underlying Zicam-mediated cell death in the nasal tissue.

The regenerative capacity of MOE will depend on the extent of necrosis. While our study showed a high degree of cellular damage into deep tissue layers of MOE, variability in clinical presentation (i.e., severity and recovery) is possible given the variable anatomy of the human nasal cavity. Indeed, published case reports indicate varying outcomes of smell dysfunction associated with Zicam use [7], [8]. Similar variations in experimental outcomes of olfactory dysfunction are also shown in animals treated with zinc sulfate [25]. The recurring theme from these studies is that various zinc-based solutions, including Zicam (at the concentration currently being marketed) are cytotoxic. Interestingly, the cytotoxic effect of Zicam on cultured human nasal tissue appears to be independent of patient disease or anatomic location, and specific to Zicam treatment. The human nasal tissues utilized in our study represent patients with varied degree of olfactory dysfunction (Table 1). The elevation of LDH and necrosis of nasal tissue was evident in all examined tissues. This finding has critical importance to hyposmic patients because Zicam use in this group could put them at a higher risk for developing anosmia than a normosmic person. Lastly, the efficacy of Zicam in treating common cold symptoms remains controversial [26], [27]. Although the adverse effects may be transient for some, the unpredictable nature of its use by the public, together with the current and previous findings [7], [8], [26] indicate that the risks of zinc-based therapy far outweigh any benefits that it may offer.

In this paper, we demonstrated the effects of several commonly used intranasal agents on olfaction using mouse and human nasal tissue. Our study is the most comprehensive examination, to date, of the role that intranasal medications have on short-term and long-term olfactory function. Intranasal administration of Zicam, unlike other tested agents, resulted in significant cytotoxicity to both mouse and human nasal tissue. This is a concerning finding given the potential development of long-lasting, and perhaps irreversible smell dysfunction. Because Zicam was previously classified as a homeopathic substance, it was not required to undergo stringent safety or efficacy evaluation as other conventional drugs. However, the Food and Drug Administration (FDA) recently issued a public health advisory cautioning against the use of some Zicam cold remedy nasal products. The FDA also issued a warning letter to the manufacturers of Zicam reclassifying these products as “drugs” that would require additional safety and efficacy testing to continue to market these products. The potential health risks of intranasal Zicam use demonstrated in our study stresses the need for stringent oversight of homeopathic remedies to protect the public from potentially unknown and dangerous side effects.

## Materials and Methods

### Animals

Adult male C57BL/6 mice (Charles River, Wilmington, MA) were used for all experiments. All work with animals was approved by the University of Washington Institutional Animal Care and Use Committee.

### Human subjects

Human subjects were recruited as a consecutive sample from a rhinology clinic at a tertiary medical center. Written consent was obtained from all subjects donating the nasal tissue. Inclusion criteria included any patient over age 18 already scheduled for an endoscopic nasal procedure. Patients were excluded if they had a history of blood borne pathogens. The recruitment of patients and the study protocol was approved by the Institutional Review Board at the University of Washington (#35031).

### Intranasal agent administration

For EOG and biochemical studies, about 15 µl of above intranasal agents were delivered slowly into the right nasal cavity of an adult mouse using a blunted 27-gauge needle. For behavioral testing, about 15 µl of intranasal agent was administered into both nasal cavities using the blunted 27-gauge needle. Animals were sedated with intraperitoneal administration of 80 mg/kg ketamine and 8 mg/kg xylazine prior to intranasal delivery of various agents.

### Electro-olfactogram (EOG)

EOG recordings from the MOE were performed as described previously [11]. Briefly, the olfactory endoturbinate was exposed by dissecting the mouse head through the septum. The recordings were performed with an agar- and saline-filled glass microelectrode in contact with apical surface of the MOE in the open circuit configuration. Odorant solutions were puffed onto the exposed epithelium for 1 second, followed by a stream of moisturized oxygen. Traces were captured and digitized using a Digidata 1200 A (Molecular Devices, Union City, CA). The traces were low-filtered at 30 Hz and sampled at 100 Hz. Multiple regions were sampled, but EOG recordings from two separate regions of endoturbinate 1 were used for the analysis. Odorants tested were isoamyl acetate (100 µm, diluted in mineral oil) and citralva (50 µm, diluted in mineral oil), vanillin (100 µm, diluted in dH_2_O), mineral oil and dH_2_O.

### Odorant habituation assay

The odorant habituation assay of adult male mice was performed as described previously [28]. The data are presented as a ratio of the number of sniffs an animal took when the odorant-laced cotton swab was first introduced to the number of sniffs observed when the water-laced swab was first introduced. Male mice urine was stored at −80C until use. All chemical odorants and male mouse urine were diluted in water.

### Tissue processing

Mouse MOE and human nasal tissues were immersed in 4% paraformaldehyde overnight at 4C, followed by cryoprotection in 30% sucrose overnight at 4C. Mouse MOE was decalcified in 0.5 M EGTA at 4C for 3–4 days prior to cryoprotection with 30% sucrose. The tissue was subsequently embedded in OCT (Sakura Finetek USA, Inc., Torrance, CA), frozen at −20C, cryosectioned at 30 µM and mounted onto slides.

### Immunofluorescence confocal microscopy and analysis

Immunofluorescence was performed as described before with the following modifications [11]. AC3 (Santa Cruz Biotechnology Inc., Santa Cruz, CA), OMP (Dako, Carpinteria, CA), and β-tubulin (Promega, Madison, WI) were used at 1∶500, 1∶5,000, and 1∶2,000 dilutions, respectively. Images were taken with Zeiss confocal microscope using either 20x or 63x objectives. AC3 immunofluorescence intensity density was measured using ImageJ software (NIH, Bethesda, MD). OMP and β-tubulin immunopositive cells were manually counted using ImageJ software in 200 µm×200 µm region. Four animals were analyzed for each intranasal agent. Both AC3 immunofluorescence intensity density, OMP and β-tubulin immunopositive cell numbers were normalized to saline-treated group.

### Hematoxylin and Eosin (H+E) staining

The tissues were processed as above and air dried for 30 minutes before staining in hematoxylin and eosin (Surgipath Medical Industries, Inc, Richmond, IL) for 1 min each. Tissues were then dehydrated in ascending series of ethanol, cleared in xylene and coverslipped with DPX (Fluka, Milwaukee, WI) and visualized with light microscope.

### Human nasal tissue biopsy

Subject disease severity was assessed with several instruments including a sinus CT scan (evaluated with Lund-Mackay staging system; LM CT) [29], nasal endoscopy and the Sino-Nasal Outcomes Test (SNOT-20) [30]. Subjects also took the University of Pennsylvania Smell Identification Test (UPSIT; Sensonics, Inc., Haddon Heights, NJ) preoperatively to assess their olfactory function. The human nasal tissue was collected during each subjects' surgery while under general anesthesia. We collected various nasal tissues from the middle turbinate, inferior turbinate or superior nasal septum based on the planned operation. The harvested nasal tissue was immediately processed as below for establishing an organotypic tissue culture system.

### Human nasal explants culture

The biopsied nasal explants were removed of any tissue debris or blood, and divided into small pieces (approximately 2–4 mm×2–4 mm×1–2 mm) using a 15-blade scalpel and a fine forcep under a dissecting scope. It was then placed on 0.4 µm polytetrafluoroethane (PTFE) membrane (Millicell-CM; Millipore, Cork, Ireland) immersed in 1.7 ml of culture medium consisting of 50% Dulbecco's Modified Eagle Medium High Glucose (Invitrogen, Carlsbad, CA); 25% Hank solution (Invitrogen, Carlsbad, CA); 50 U/mL penicillin G and 40 µg/mL streptomycin (Invitrogen, Carlsbad, CA) in a 60×15 mm tissue culture dish (Corning, Corning, NY). The tissue was placed on the membrane such that epithelial side was exposed to the air, and placed in humidified incubator at 37°C with 5% CO_2_. The culture media was changed every day.

### Lactate dehydrogenase (LDH) assay

LDH assay was performed with CytoTox 96 Non-Radioactive Cytotoxicity Assay Kit (Promega, Madison, WI) with the following modifications. On the day of the assay, 50 µl of culture medium was removed and added to 50 µl of ‘substrate mix’. Following 30 minute incubation at room temperature, the reaction was stopped with 50 µl of ‘stop solution’. Absorbance at 490 nm was obtained with Epx Precision Microplate Reader (Molecular Devices, Sunnyvale, CA). For measuring LDH levels in the human nasal explants, we stabilized the nasal tissue in the cultured environment for approximately 48 hours. We then measured the baseline LDH levels, and applied 2 µl of either 0.9% saline, Afrin (Schering-Plough HealthCare Products, Inc., Kenilworth, NJ), Nasacort (Sanofi-Aventis, Bridgewater, NJ), lidocaine (Hospira, Inc., Lake Forest, IL), 1∶100,000 epinephrine (diluted with 0.9% saline; Hospira, Inc., Lake Forest, IL) or Zicam (Matrixx Initiatives, Inc., Phoenix, AZ) directly onto the tissue. The levels of LDH were again measured 24 hours later. We repeated the addition of the intranasal agent and LDH measurement for five days.

### Statistical analysis

Data are reported as mean +/− S.E.M. We performed statistical analyses using the two-tailed Student t-test. Statistical significance was defined as P<0.05. For the human tissue analysis, a power calculation was performed that showed a sample size of three would give 80% power to detect a significant difference (based on two pilot samples that showed a large effect size). We used linear mixed regression modeling to account for the repeated measures of the LDH assays on successive days for each tissue sample. To account for multiple comparisons, we used Bonferroni adjusted P-values to determine statistical significance (P = 0.05/25 = 0.002 for significance).

## Supporting Information

Figure S1 Gross appearance of mouse MOE 9 days after various intranasal agent administrations. Note the atrophy of MOE in Zicam-treated mouse. Black arrowheads indicate atrophic endoturbinates.
(3.15 MB TIF)

Figure S2 Damage to mouse olfactory epithelium following Zicam treatment at various time points. H&E staining of mouse MOE depicting a significant loss of epithelium and submucosal damage 9 days after intranasal administration of Zicam as compared to saline treatment. Much greater damages to the epithelium and submucosal structure are observed without evidence of regeneration 31 and 35 days after intranasal administration of Zicam. Black arrows indicate damaged and remnants of MOE with fibrosis (e.g., days 31 and 65) in Zicam-treated mice. Scale bar, 100 µm.
(2.94 MB TIF)

Figure S3 Cell death in human nasal explants following Zicam treatment from various regions of nasal cavity. H&E staining is shown. (a) Subject 1, inferior turbinate. (b) Subject 2, middle turbinate. (c) Subject 3, superior nasal septum. (d) Subject 4, middle turbinate.
(2.87 MB TIF)
