# Supplementary figures and images for: Zicam-Induced Damage to Mouse and Human Nasal Tissue
Source: PLoS One. 2009 Oct 30;4(10):e7647. doi: 10.1371/journal.pone.0007647 (PMC2765727; doi:10.1371/journal.pone.0007647)

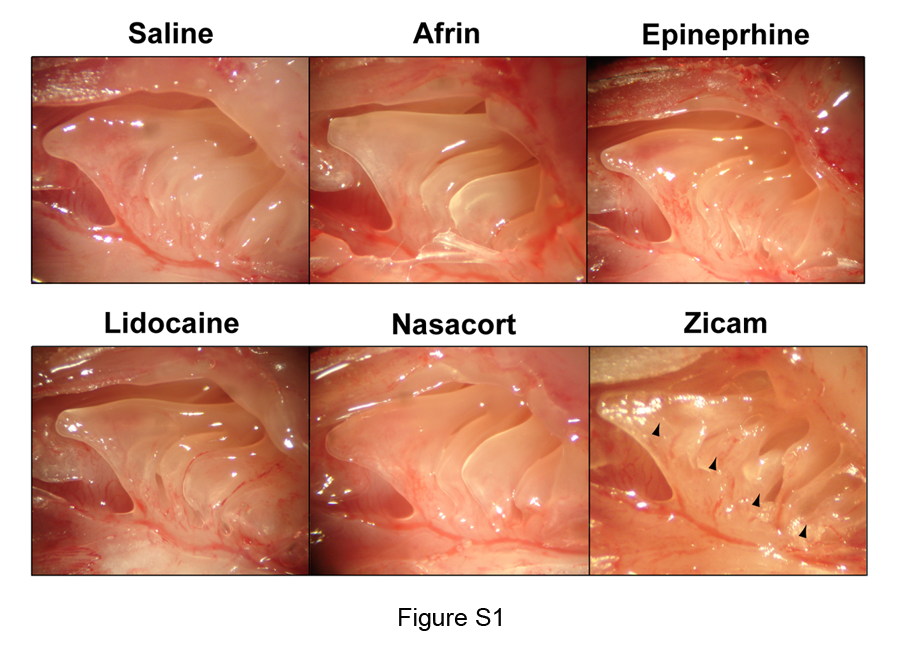

Supplement: Figure S1 — Gross appearance of mouse MOE 9 days after various intranasal agent administrations. Note the atrophy of MOE in Zicam-treated mouse. Black arrowheads indicate atrophic endoturbinates. (3.15 MB TIF) [file pone.0007647.s001.tif]

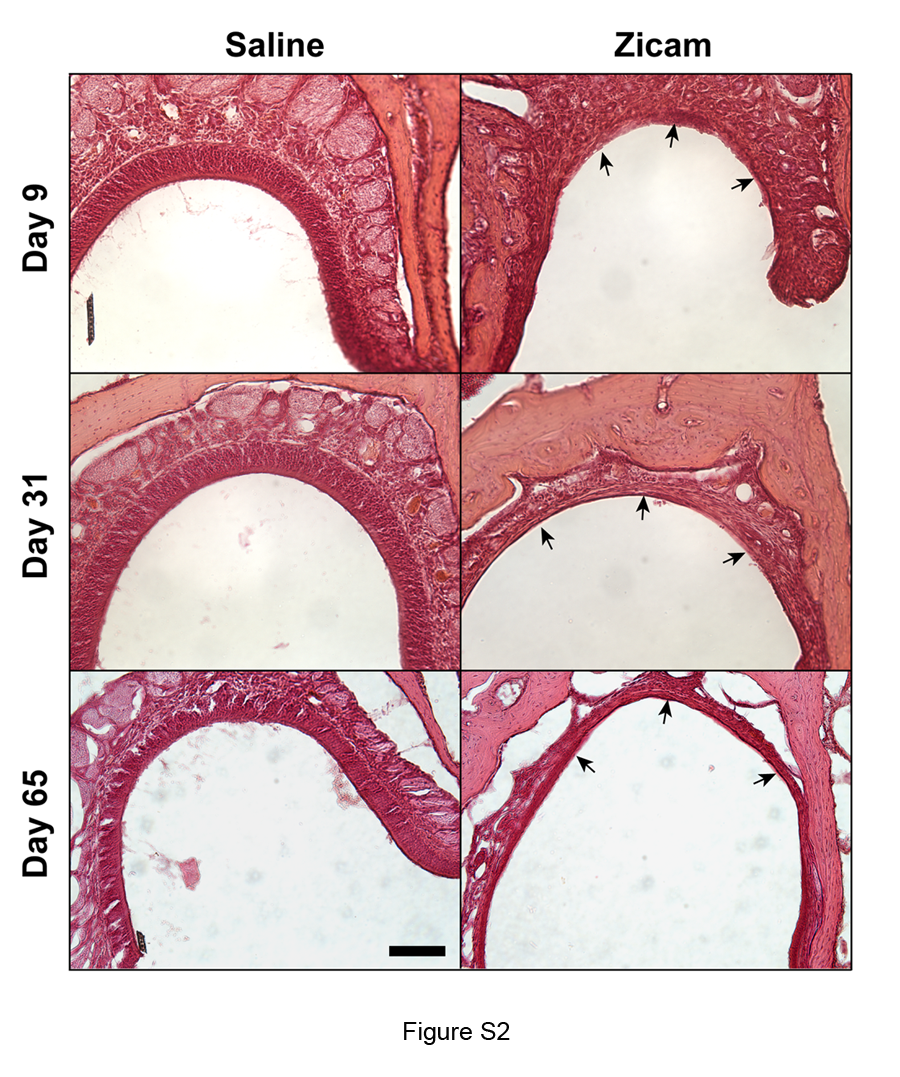

Supplement: Figure S2 — Damage to mouse olfactory epithelium following Zicam treatment at various time points. H&E staining of mouse MOE depicting a significant loss of epithelium and submucosal damage 9 days after intranasal administration of Zicam as compared to saline treatment. Much greater damages to the epithelium and submucosal structure are observed without evidence of regeneration 31 and 35 days after intranasal administration of Zicam. Black arrows indicate damaged and remnants of MOE with fibrosis (e.g., days 31 and 65) in Zicam-treated mice. Scale bar, 100 µm. (2.94 MB TIF) [file pone.0007647.s002.tif]

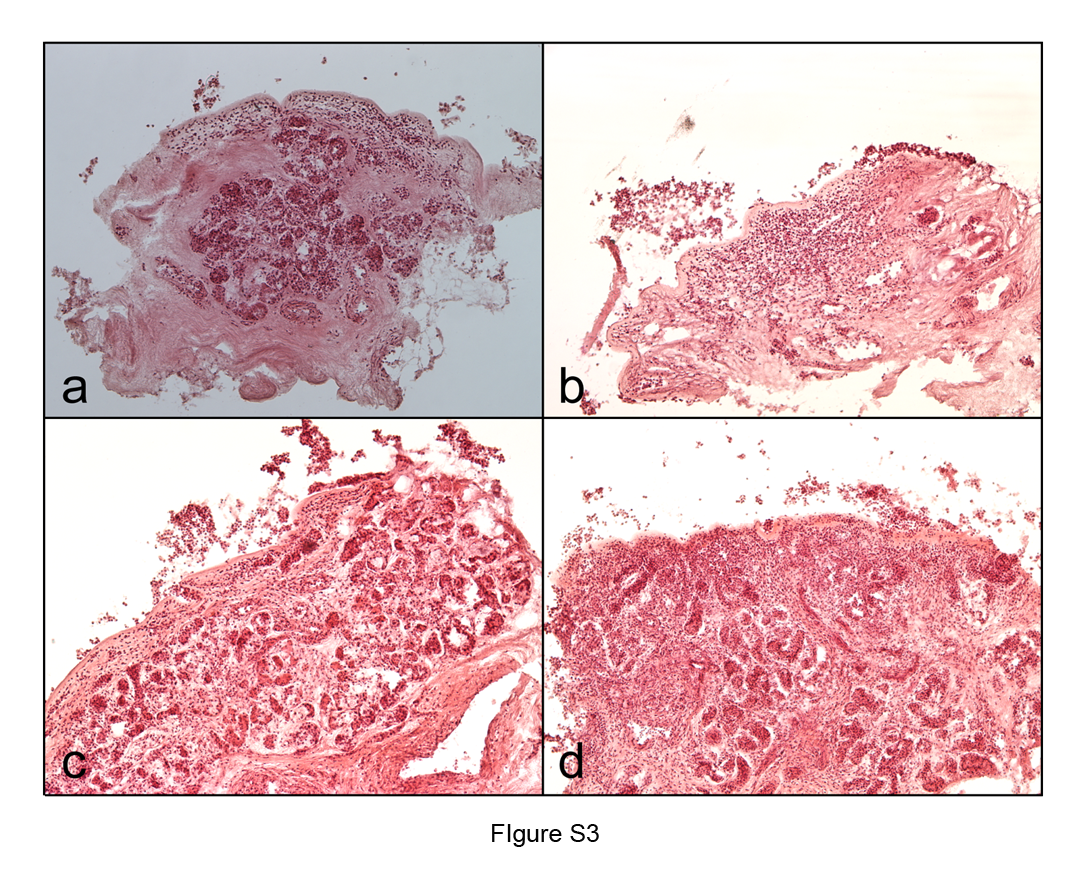

Supplement: Figure S3 — Cell death in human nasal explants following Zicam treatment from various regions of nasal cavity. H&E staining is shown. (a) Subject 1, inferior turbinate. (b) Subject 2, middle turbinate. (c) Subject 3, superior nasal septum. (d) Subject 4, middle turbinate. (2.87 MB TIF) [file pone.0007647.s003.tif]
